# Epidemiological research priorities for public health control of the ongoing global novel coronavirus (2019-nCoV) outbreak

**DOI:** 10.2807/1560-7917.ES.2020.25.6.2000110

**Published:** 2020-02-13

**Authors:** Benjamin J Cowling, Gabriel M Leung

**Affiliations:** 1 WHO Collaborating Centre for Infectious Disease Epidemiology and Control, School of Public Health, The University of Hong Kong, Hong Kong Special Administrative Region, China

**Keywords:** 2019-nCoV, coronavirus, emerging infections, response, air-borne infections, viral infections, emerging or re-emerging diseases

It is now 6 weeks since Chinese health authorities announced the discovery of a novel coronavirus (2019-nCoV) [1] causing a cluster of pneumonia cases in Wuhan, the major transport hub of central China. The earliest human infections had occurred by early December 2019, and a large wet market in central Wuhan was linked to most, but not all, of the initial cases [2]. While evidence from the initial outbreak investigations seemed to suggest that 2019-nCoV could not easily spread between humans [3], it is now very clear that infections have been spreading from person to person [2]. We recently estimated that more than 75,000 infections may have occurred in Wuhan as at 25 January 2020 [4], and increasing numbers of infections continue to be detected in other cities in mainland China and around the world. A number of important characteristics of 2019-nCoV infection have already been identified, but in order to calibrate public health responses we need improved information on transmission dynamics, severity of the disease, immunity, and the impact of control and mitigation measures that have been applied to date.

## Transmission dynamics

Infections with 2019-nCoV can spread from person to person, and in the earliest phase of the outbreak the basic reproductive number was estimated to be around 2.2, assuming a mean serial interval of 7.5 days [2]. The serial interval was not precisely estimated, and a potentially shorter mean serial interval would have corresponded to a slightly lower basic reproductive number. Control measures and changes in population behaviour later in January should have reduced the effective reproductive number. However, it is too early to estimate whether the effective reproductive number has been reduced to below the critical threshold of 1 because cases currently being detected and reported would have mostly been infected in mid- to late-January. Average delays between infection and illness onset have been estimated at around 5–6 days, with an upper limit of around 11-14 days [2,5], and delays from illness onset to laboratory confirmation added a further 10 days on average [2].

Chains of transmission have now been reported in a number of locations outside of mainland China. Within the coming days or weeks it will become clear whether sustained local transmission has been occurring in other cities outside of Hubei province in China, or in other countries. If sustained transmission does occur in other locations, it would be valuable to determine whether there is variation in transmissibility by location, for example because of different behaviours or control measures, or because of different environmental conditions. To address the latter, virus survival studies can be done in the laboratory to confirm whether there are preferred ranges of temperature or humidity for 2019-nCoV transmission to occur.

In an analysis of the first 425 confirmed cases of infection, 73% of cases with illness onset between 12 and 22 January reported no exposure to either a wet market or another person with symptoms of a respiratory illness [2]. The lack of reported exposure to another ill person could be attributed to lack of awareness or recall bias, but China’s health minister publicly warned that pre-symptomatic transmission could be occurring [6]. Determining the extent to which asymptomatic or pre-symptomatic transmission might be occurring is an urgent priority, because it has direct implications for public health and hospital infection control. Data on viral shedding dynamics could help in assessing duration of infectiousness. For severe acute respiratory syndrome-related coronavirus (SARS-CoV), infectivity peaked at around 10 days after illness onset [7], consistent with the peak in viral load at around that time [8]. This allowed control of the SARS epidemic through prompt detection of cases and strict isolation. For influenza virus infections, virus shedding is highest on the day of illness onset and relatively higher from shortly before symptom onset until a few days after onset [9]. To date, transmission patterns of 2019-nCoV appear more similar to influenza, with contagiousness occurring around the time of symptom onset, rather than SARS.

Transmission of respiratory viruses generally happens through large respiratory droplets, but some respiratory viruses can spread through fine particle aerosols [10], and indirect transmission via fomites can also play a role. Coronaviruses can also infect the human gastrointestinal tract [11,12], and faecal-oral transmission might also play a role in this instance. The SARS-CoV superspreading event at Amoy Gardens where more than 300 cases were infected was attributed to faecal-oral, then airborne, spread through pressure differentials between contaminated effluent pipes, bathroom floor drains and flushing toilets [13]. The first large identifiable superspreading event during the present 2019-nCoV outbreak has apparently taken place on the *Diamond Princess* cruise liner quarantined off the coast of Yokohama, Japan, with at least 130 passengers tested positive for 2019-nCoV as at 10 February 2020 [14]. Identifying which modes are important for 2019-nCoV transmission would inform the importance of personal protective measures such as face masks (and specifically which types) and hand hygiene.

## Disease severity and immunity

The first human infections were identified through a surveillance system for pneumonia of unknown aetiology, and all of the earliest infections therefore had pneumonia. It is well established that some infections can be severe, particularly in older adults with underlying medical conditions [15,16], but based on the generally mild clinical presentation of 2019-nCoV cases detected outside China, it appears that there could be many more mild infections than severe infections. Determining the spectrum of clinical manifestations of 2019-nCoV infections is perhaps the most urgent research priority, because it determines the strength of public health response required. If the seriousness of infection is similar to the 1918/19 Spanish influenza, and therefore at the upper end of severity scales in influenza pandemic plans, the same responses would be warranted for 2019-nCoV as for the most severe influenza pandemics. If, however, the seriousness of infection is similar to seasonal influenza, especially during milder seasons, mitigation measures could be tuned accordingly.

Beyond a robust assessment of overall severity, it is also important to determine high risk groups. Infections would likely be more severe in older adults, obese individuals or those with underlying medical conditions, but there have not yet been reports of severity of infections in pregnant women, and very few cases have been reported in children [2].

Those under 18 years are a critical group to study in order to tease out the relative roles of susceptibility vs severity as possible underlying causes for the very rare recorded instances of infection in this age group. Are children protected from infection or do they not fall ill after infection? If they are naturally immune, which is unlikely, we should understand why; otherwise, even if they do not show symptoms, it is important to know if they shed the virus. Obviously, the question about virus shedding of those being infected but asymptomatic leads to the crucial question of infectivity. Answers to these questions are especially pertinent as basis for decisions on school closure as a social distancing intervention, which can be hugely disruptive not only for students but also because of its knock-on effect for child care and parental duties. Very few children have been confirmed 2019-nCoV cases so far but that does not necessarily mean that they are less susceptible or that they could not be latent carriers. Serosurveys in affected locations could inform this, in addition to truly assessing the clinical severity spectrum.

Another question on susceptibility is regarding whether 2019-nCoV infection confers neutralising immunity, usually but not always, indicated by the presence of neutralising antibodies in convalescent sera. Some experts already questioned whether the 2019-nCoV may behave similarly to MERS-CoV in cases exhibiting mild symptoms without eliciting neutralising antibodies [17]. A separate question pertains to the possibility of antibody-dependent enhancement of infection or of disease [18,19]. If either of these were to be relevant, the transmission dynamics could become more complex.

## Control and mitigation measures

A wide range of control measures can be considered to contain or mitigate an emerging infection such as 2019-nCoV. Internationally, the past week has seen an increasing number of countries issue travel advisories or outright entry bans on persons from Hubei province or China as a whole, as well as substantial cuts in flights to and from affected areas out of commercial considerations. Evaluation of these mobility restrictions can confirm their potential effectiveness in delaying local epidemics [20], and can also inform when as well as how to lift these restrictions.

If and when local transmission begins in a particular location, a variety of community mitigation measures can be implemented by health authorities to reduce transmission and thus reduce the growth rate of an epidemic, reduce the height of the epidemic peak and the peak demand on healthcare services, as well as reduce the total number of infected persons [21]. A number of social distancing measures have already been implemented in Chinese cities in the past few weeks including school and workplace closures. It should now be an urgent priority to quantify the effects of these measures and specifically whether they can reduce the effective reproductive number below 1, because this will guide the response strategies in other locations. During the 1918/19 influenza pandemic, cities in the United States, which implemented the most aggressive and sustained community measures were the most successful ones in mitigating the impact of that pandemic [22].

Similarly to international travel interventions, local social distancing measures should be assessed for their impact and when they could be safely discontinued, albeit in a coordinated and deliberate manner across China such that recrudescence in the epidemic curve is minimised. Mobile telephony global positioning system (GPS) data and location services data from social media providers such as Baidu and Tencent in China could become the first occasion when these data inform outbreak control in real time.

At the individual level, surgical face masks have often been a particularly visible image from affected cities in China. Face masks are essential components of personal protective equipment in healthcare settings, and should be recommended for ill persons in the community or for those who care for ill persons. However, there is now a shortage of supply of masks in China and elsewhere, and debates are ongoing about their protective value for uninfected persons in the general community.

The Table summarises research gaps to guide the public health response identified.

**Table:** Research priorities to guide the public health response to 2019-nCoV

Domain	Priorities	Study designs / data sources required
Transmission dynamics	Provide robust estimates of the serial interval and generation time	Detailed exposure and illness onset information from unselected case clusters in line lists, preferably from more than one epicentre
	Estimate effective reproductive number (*R_t_*) in other cities (i.e. ex-Wuhan) in China and elsewhere	Epidemic curves for each city by dates of illness onset, preferably stratified by likely source of infection (zoonotic, environmental point source, local case vs imported index case)
	Clarify the relative importance of pre-symptomatic / asymptomatic transmission	Detailed reports of transmission events and symptomatic status of infectors; viral shedding data; special studies in households and other closed settings
	Determine the role of different age groups in transmission, particularly children	Transmission studies in households and other closed settings; serological studies
	Determine the relative importance of possible modes of transmission	Outbreak investigations, in particular for superspreading events; environmental sampling, air sampling and exhaled breath sampling; special studies in households and other closed settings
	Determine environmental effects on virus survival and transmission	Virus survival studies in situ vivo and in vitro; environmental sampling studies

Severity	Provide robust estimates of the risk of fatality of hospitalised cases, by age or other important groupings	Reports from unselected clinical cohorts of times to death or recovery among resolved cases
	Provide robust estimates of the risk of fatality of symptomatic cases, by age or other important groupings	Estimates of incidence from population-wide surveillance of mild cases
	Identify groups at high risk of severe infection	Case–control studies; cohort studies

Susceptibility	Determine if children are infected, and if so, if they are infectious	Transmission studies in households and other closed settings; serological studies
	Determine if all infections result in neutralising immunity	Convalescent serology from mild as well as severe cases, in all age groups

Control measures	Provide impact estimates of travel restrictions, border screening and quarantine policies on non-local spread	Modelling analyses of local and global spread of infections
	Estimate the effects of social distancing measures and other non-pharmaceutical interventions on transmissibility	Comparative analyses of transmissibility in different locations
	Predict the most effective measures to reduce the peak burden on healthcare providers and other societal functions	Modelling studies incorporating healthcare capacity and processes

In conclusion, there are a number of urgent research priorities to inform the public health response to the global spread of 2019-nCoV infections. Establishing robust estimates of the clinical severity of infections is probably the most pressing, because flattening out the surge in hospital admissions would be essential if there is a danger of hospitals becoming overwhelmed with patients who require inpatient care, not only for those infected with 2019-nCoV but also for urgent acute care of patients with other conditions including those scheduled for procedures and operations. In addressing the research gaps identified here, there is a need for strong collaboration of a competent corps of epidemiological scientists and public health workers who have the flexibility to cope with the surge capacity required, as well as support from laboratories that can deliver on the ever rising demand for diagnostic tests for 2019-nCoV and related sequelae. The readiness survey by Reusken et al. in this issue of *Eurosurveillance* testifies to the rapid response and capabilities of laboratories across Europe should the outbreak originating in Wuhan reach this continent [23].

In the medium term, we look towards the identification of efficacious pharmaceutical agents to prevent and treat what may likely become an endemic infection globally. Beyond the first year, one interesting possibility in the longer term, perhaps borne of wishful hope, is that after the first few epidemic waves, the subsequent endemic re-infections could be of milder severity. Particularly if children are being infected and are developing immunity hereafter, 2019-nCoV could optimistically become the fifth human coronavirus causing the common cold.

## Editorial note

This editorial was published as an e-Alert on 11 February 2020.
